# A relevant long-term impact of the circulation of a potentially contaminated vaccine on the distribution of scrapie in Italy. Results from a retrospective cohort study

**DOI:** 10.1186/1297-9716-43-63

**Published:** 2012-08-28

**Authors:** Silvia Bertolini, Cristiana Maurella, Cristina Bona, Francesco Ingravalle, Rosanna Desiato, Elisa Baioni, Laura Chiavacci, Maria Caramelli, Giuseppe Ru

**Affiliations:** 1 Epidemiologia e Osservatorio Epidemiologico, Istituto Zooprofilattico Sperimentale del Piemonte, Liguria e Valle d'Aosta (IZSPLVA), Via Bologna 148, 10154, Turin, Italy

## Abstract

A sudden increase in the incidence of scrapie in Italy in 1997 was subsequently linked to the use of a potentially infected vaccine against *contagious agalactia*. The relative risk for the exposed farms ranged between 6 and 40. The aim of this study was to assess the long-term impact of exposure to the potentially scrapie-contaminated vaccine on the Italian classical scrapie epidemic. We carried out a retrospective cohort study, fitting mixed-effects Poisson regression models, dividing national geographic areas into exposure categories on the basis of the vaccine circulation levels. We took into account the sensitivity of the surveillance system applied in the different areas. The population attributable fraction (PAF) was used to assess the impact on the total population of farms associated with the effect of circulation of the vaccine. The provinces where the vaccine was more often sold were noted to have a higher level of disease when compared to those provinces where the vaccine was sold less often (incidence rate ratio [IRR]: 2.7; 95% confidence interval [CI]: 1.1-6.5). The population attributable fraction was high (68.4%). Standardization techniques allowed to account for the potential of geographical variability in the sensitivity of the Italian surveillance system. Although the number of the directly exposed farms was limited, an important long-term impact of the vaccine circulation could be quantified in terms of secondary outbreaks likely due to the exchange of animals from directly exposed flocks.

## Introduction

A sudden increase in the incidence of scrapie in Italy in 1997 was subsequently linked to the administration of a potentially infected vaccine against *contagious agalactia*. The commonly known risk factors for scrapie are the prion protein genotype and flock management practices such as movement of animals, sharing pastures with other flocks, and flock size [1-13]. As the epidemic unfolded, the large number of cases, the temporal clustering of the outbreaks, the exceptional involvement of goats, a species in which scrapie is rarely reported, the high in-flock incidence and the recurring reporting of exposure to a unique vaccine in many outbreaks all pointed to an accidental infection [14]. This hypothesis was consistent with Gordon’s description (1946) of a similar episode that had occurred in Scotland in 1937 when a sudden epidemic peak of scrapie was associated with the circulation of a vaccine against Louping ill. The investigations led to a vaccine batch that had been produced from the nervous tissue of lambs affected by scrapie in the incubative stage of the infection [15].

In the Italian accident, the vaccine was a formol-inactivated immunogen against *contagious agalactia* prepared by a single manufacturer (Rome, Italy) from the brain and mammary gland homogenates of sheep experimentally infected with *Mycoplasma agalactiae*[14,16]. The vaccine was distributed between November 1994 and December 1996 in eight regions of central and southern Italy where flocks currently number about 78 000.

A study conducted in 2004 by our group has shown that the relative risk for the exposed farms versus the farms where the animals had not been administered the vaccine ranged between 6 and 40. However, the impact on the general population in terms of proportion of disease attributable to exposure was limited, probably because of the limited number of the farms that were directly exposed [17]. The effect the circulation of a potentially infected vaccine may have on the onset of secondary outbreaks and its effect in terms of population impact can be quantified by analysing long-term data series.

In European countries the passive surveillance system for scrapie has been integrated since 2002 with an active surveillance program targeting samples of the sheep and goat populations older than 18 months of age. Animals are identified within different risk streams, i.e., as healthy animals at abattoir or fallen stock, and are submitted to the so-called rapid tests for the detection of abnormal PrP in the brainstem. The probability of case detection (i.e., surveillance sensitivity) is highest among fallen stock and increases with the number of animals tested per flock [18-21]. Accordingly, standardized incidence data adjusted for surveillance sensitivity at the provincial level are needed in order to make unbiased geographical comparisons.

The application of surveillance systems with geographical heterogeneous sensitivity may lead to different probabilities to detect the disease. Observed incidence rates or prevalence data don't take these differences into account and could be misleading when making geographical comparisons.

The aim of this study was to assess the long-term impact of exposure to a potentially scrapie-contaminated vaccine on the Italian classical scrapie epidemic. We carried out a retrospective cohort study, dividing national geographic areas into exposure categories on the basis of the vaccine circulation levels. We compared the disease frequency in the subsequent years in each of the exposure categories, taking into account the sensitivity of the surveillance system applied.

In this way we were able to identify an association between the surveillance-adjusted incidence level of the disease and the diffusion of the vaccine even long after direct exposure. Also, we were able to quantify the impact in terms of percentage of outbreaks attributable to the circulation of the vaccine by estimating the population attributable fraction.

## Materials and methods

### Study area

The analysis focused on the eight regions of central and southern Italy where the vaccine had been distributed (Tuscany, Lazio, Campania, Apulia, Basilicata, Calabria, Sicily and Sardinia). The vaccine had been reportedly used in 29 out of 45 provinces in these regions.

### Databases

The data from three different datasets were analysed: national surveillance data, vaccine distribution data and National Animal Registry Office data.

#### National Surveillance data

We used data from the Small Ruminant TSE national database maintained by the Reference Centre for Animal Encephalopathy at IZSPLVA.

Data from active surveillance for scrapie in sheep and goats in the study area from 1 January 2002 to 31 December 2005^a^ were considered because we assumed that data from later years could conceal the potential effect of the vaccine. This dataset comprised data from 95 714 animals tested in the surveillance stream of regularly slaughtered animals and from 11 757 animals tested in the fallen stock stream, thus recruiting a total of 16 755 flocks for the study. These data were then aggregated at the flock level to obtain the number of animals tested per holding in the two surveillance streams. County, parish, and holding (CPH) identifiers were used to identify each flock.

We then used the data on the 50 outbreaks of classical scrapie in sheep and 7 outbreaks in goats identified by active surveillance on fallen stock and regularly slaughtered animals in the study area during the same period.

#### Vaccine distribution data

A list was obtained of the 177 flock farmers who had bought the formol inactivated vaccine against *contagious agalactia* between November 1994 and December 1996. This period is consistent with the higher risk of contamination of the vaccine according to the information provided by the vaccine manufacturer. Although brain homogenates of sheep infected with *Mycoplasma agalactiae* were no longer used as an ingredient in the vaccine, its production continued up to July 1997. Not all the potentially exposed flocks could be traced back, however, because in some cases the list included only the name of a veterinarian or a retailer instead of the flock farmer’s name. To fill this gap, we compiled a list of 29 veterinarians or retailers who had purchased the vaccine as own stock during the period in question. Data from the two lists were aggregated to provide provincial level data.

#### National Animal Registry Office data

We considered the number of farms registered within the National Animal Registry Office for each province. In order to obtain a more reliable size of the susceptible population we decided to use the average number of farms in operation in the 8 regions (78 463 farms) during the 2006–2009 period obtained from the identification and registration system that went into effect at the national level in 2005.

### Statistical analysis

Stata Software 11.1 (Stata Corp, College Station, TX) was used to create the databases and process the data for the statistical analysis. The ArcGIS version 9.2 (ESRI, Redlands, CA, USA) was used to create the thematic maps.

In this retrospective cohort study the incidence rates for each province for the entire study period were calculated as the ratio of the number of new outbreaks observed per 1000 registered farms.

Direct standardization techniques [23,24] were applied to adjust for surveillance sensitivity in the eight provinces. The incidence rates, standardized on the applied surveillance, allow to obtain the distribution of the disease as if the surveillance was uniformly applied throughout the entire area. We used the *dstdize* command (Stata).

The total number of farms registered with the National Animal Registry Office in the 108 Italian provinces (*n* = 138 721) was used as external standard population, from which nine levels of surveillance sensitivity were created based on both the surveillance stream (regularly slaughtered and fallen stock categories) and the categorization of the number of animals tested by stream on each farm.

Within each surveillance stream we defined three sensitivity levels: for the regularly-slaughtered category, a level of 1, 2 or 3 was applied when 1 to 5 (75% of farms), 6 to 12 (15% of farms) or 13 or more animals (10% of farms), respectively, had been tested. Additionally, the farms where no regularly-slaughtered animals had been tested were included into level 1, accounting for the proportion of farms never involved by active surveillance. For the fallen stock stream, the respective intervals were 1 to 2 (75% of farms), 3 to 5 (15% of farms), and 6 or more animals (10% of farms); again, the farms where no fallen stock animals had been tested were included into level 1. Table 1 illustrates the scheme of the various combinations.

**Table 1:** Distribution of Italian farms of small ruminants according to the levels of surveillance sensitivity

**Global surveillance sensitivity level**	**Level 1 Surveillance Sensitivity**	**Level 2 Surveillance Sensitivity**	**Number of farms**
	**(based on the number of regularly slaughtered animals)**	**(based on the number of fallen stock animals)**	
1	1	1	131 893
2	1	2	1157
3	1	3	585
4	2	1	2884
5	2	2	101
6	2	3	74
7	3	1	1862
8	3	2	75
9	3	3	90

This distribution was used as external standard population to calculate the standardized incidence rates. For both variables, the classes were created on the basis of the distribution of number of farms (level 1: 75% of farms; level 2: 15% of farms; level 3: 10% of farms. Additionally, the farms where no regularly slaughtered or fallen stock animals had been tested were respectively included into level 1).

Most of the total number of the farms (95.08% of the 138 721 registered farms) were categorized as global surveillance sensitivity level 1.

To estimate the association between vaccine exposure and disease levels for each province, we carried out a preliminary univariate analysis based on Poisson models, wherein the relative risks were computed to obtain the incidence rate ratios. In order to consider the herd size as a possible confounder we included the median of the herd size for each province in this analysis. We then fitted mixed-effects Poisson regression models including the variables that resulted as having a health impact from the univariate analysis. We used a *xtpoisson* command (Stata) because in this function a gamma distribution with mean 1 and variance alpha is assumed for the level-2 random intercept, to account for a possible within-cluster dependence (i.e. overdispersion) [25].

The number of farms in a given province that had purchased the vaccine was used to define an explanatory variable to quantify the vaccine circulation. In a first model, the number of the observed outbreaks for each province was entered as a dependent variable to assess the role of exposure to vaccine. The number of registered flocks in each province was considered as offset. Region was included as a random-effect variable.

To investigate the potential role of the vaccine due to its distribution through veterinarians or retailers, a categorical variable based on the number of veterinarians or retailers who had purchased the vaccine as own stock in each province was subsequently added as a covariate.

On the basis of the number of farms which had bought the vaccine, three exposure levels (EL_farm_) were defined: (1) provinces where 0–4 farms bought the vaccine (66.7% of provinces); (2) provinces where 5–10 farms bought the vaccine (20.0% of provinces); (3) provinces where > 10 farms bought the vaccine (13.3% of provinces). For the variable based on the number of veterinarians or retailers, two exposure levels (EL_vet_) were defined: (1) provinces where 0–1 veterinarian or retailer bought the vaccine (86.7% of provinces); (2) provinces where > 1 veterinarian or retailer bought the vaccine (13.3% of provinces).

The Akaike Information Criterion (AIC) was applied to compare the models including and not including each explanatory variable and to compare the mixed- and fixed-effect models [26]. These models were then fitted using standardized incidence rates adjusted for surveillance sensitivity based on the data shown in Table 1. The number of expected outbreaks for each province constituted the dependent variable obtained by multiplying the standardized rates by the number of registered farms.

The statistical significance of a linear dose-response trend of the number of farms which had bought the vaccine (exposure level) with respect to the disease incidence level was tested using the Wald test to verify the statistical significance of the linear regression coefficient of the vaccine circulation variable entered as a continuous variable in all four models [27,28].

Finally, the population attributable fraction (PAF) was used to assess the impact on the total population of farms associated with the circulation of the vaccine. PAF is the proportion of disease among the total population that would be eliminated if the exposure were eliminated [29]. In this case, we applied the formula suggested by Rockhill because we had multicategory exposures and the presence of confounding using model 3 results on the basis of the AIC value and model coefficients [30]:

(1) $$\sum_{i-o}^{k}pd_{i}\left(\frac{PR_{i}-1}{RR_{i}}\right)=1-\sum_{i-o}^{k}\frac{pd_{i}}{RP_{i}}$$

where

pd_i_ is the proportion of total cases in the population arising from the *i*th exposure category;

RR_i_ is the adjusted relative risk for the *i*th exposure category (relative to the unexposed stratum).

### Graphic representation

The distribution of exposure to the vaccine and the distribution of disease in the province where the vaccine had been distributed are shown in thematic maps. The maps were generated from the number of farms that had purchased the vaccine and the standardized rates for each of the 45 provinces.

## Results

Twenty-five provinces of the 8 regions where the vaccine had been distributed experienced at least one scrapie outbreak. The range of variability of the 4-year standardized incidence rates (2002–2005) for the 25 provinces was 0.20 to 5.8 outbreaks/1000 farms, with ≤ 0.84 cases/1000 farms in 50% of these provinces.

**Figure 1:**
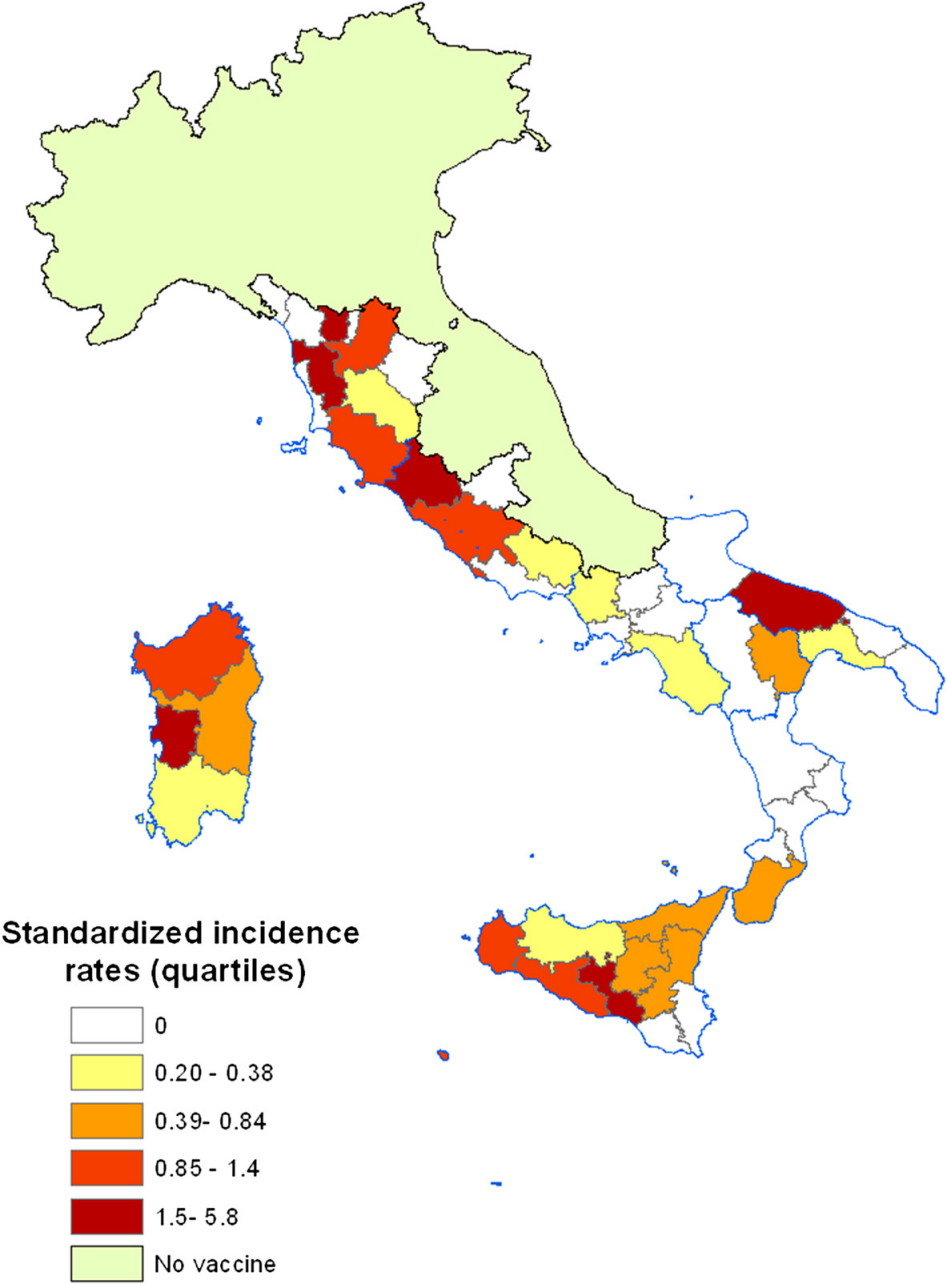
**Standardized incidence rates in the provinces of the regions where the vaccine circulated (quartile).** The provinces characterized by the highest risk of disease are concentrated in central Italy, Sicily and Sardinia.

Figure 1 shows the distribution by province of the standardized incidence rates in the 8 regions where the vaccine circulated: the provinces with the highest risk of the disease are concentrated in central Italy, Sicily and Sardinia.

**Figure 2:**
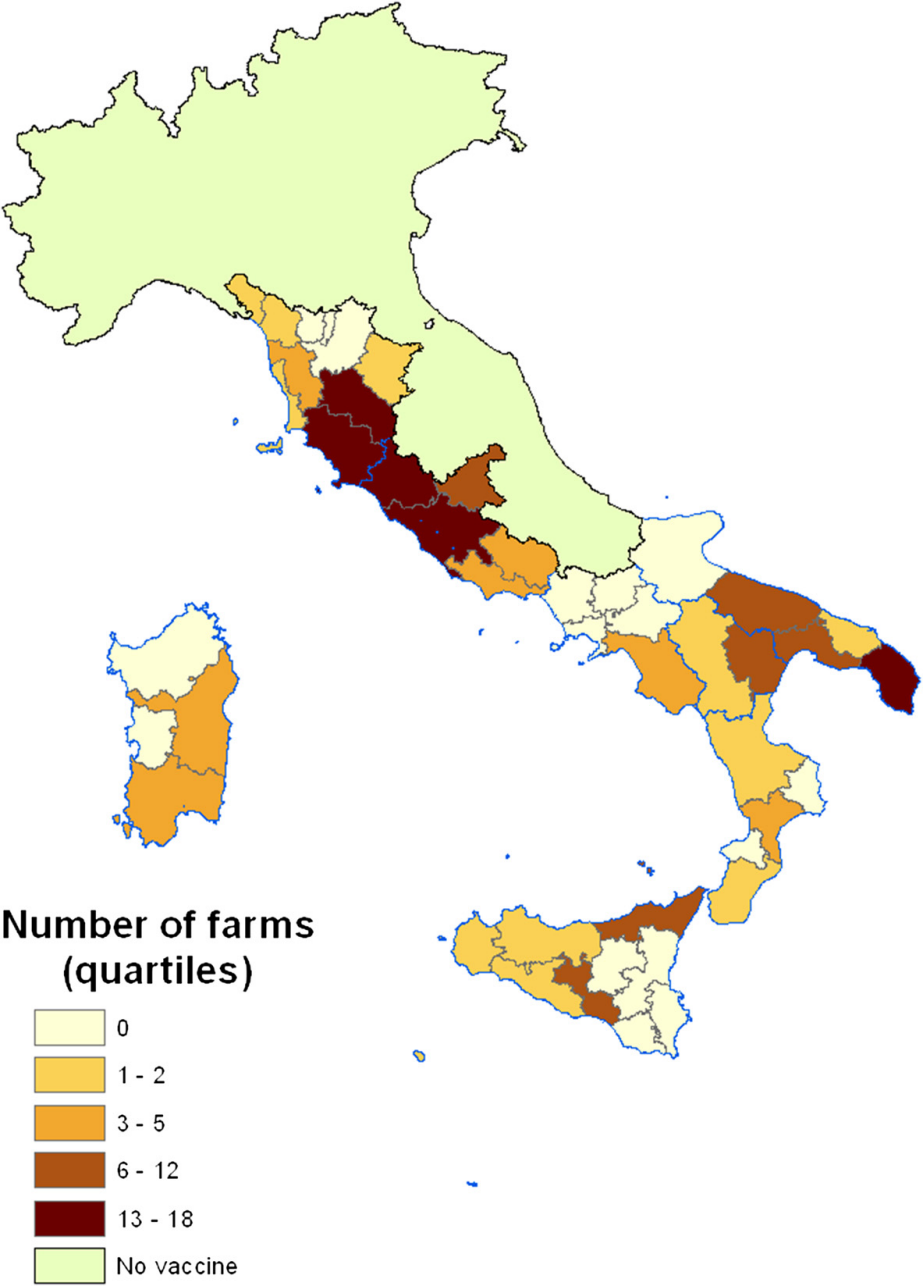
**Distribution of the number of purchaser farms in the eight Italian regions (quartile).** The map shows the distribution of the number of farms that had bought the vaccine directly from the manufacturer between November 1994 and December 1996 in each province. Most of the provinces where an elevated number of farms had purchased the vaccine are concentrated in central Italy.

Figure 2 illustrates the distribution by province of the number of farms that had directly purchased the vaccine from the manufacturer between November 1994 and December 1996 in the 8 regions: most of the provinces where an elevated number of farms purchased the vaccine are concentrated in central Italy. In 29 of the 45 provinces at least one farm had directly bought the vaccine. In 50% of the 29 provinces less than 6 farms had purchased the vaccine (range: 1–18). In 16 provinces the vaccine was also distributed by one or more retailers or veterinarians.

As both the exposure variables (i.e. categorization of the number of veterinarians or retailers who had purchased the vaccine and categorization of the number of farms that had bought the vaccine) showed a significant association at the univariate analysis, they were considered as potential risk factors (Table 2) to be entered into the subsequent regression models. Even the median farm size per province was statistically significant in the preliminary analysis; however, when included in the final models, it didn't have an impact on the effect of the other variables (data not shown).

**Table 2:** Results of the univariate analysis for the vaccine exposure variables levels (IRR = incidence rate ratio)

	**Exposure level**	**Total no. of flocks**	**No. of positive flocks**	**Incidence**	**IRR**
				**(new cases per 1000 farms)**	**(95%CI)**
Categorization of the number of vets or retailers who had bought the vaccine	1 (0-1vet or retailer)	71 216	47	0.66	Referent
				
	2 (>1 vet or retailer)	7247	10	1.4	2.1 (1.1-4.1)
				
Categorization of the number of farms that had bought the vaccine	1 (0–4 farms)	49 849	25	0.50 (*)	Referent
					
	2 (5–10 farms)	20 821	17	0.82 (*)	1.6 (0.88-3.0)
				
	3 (>10 farms)	7793	15	1.9 (*)	3.8 (2.0-7.3)
				
Herd size (median for each province)		78 463	57		1.005
					(1.001-1.01)

Both exposure variables related to the vaccine [(1) categorization of the number of veterinarians or retailers who had purchased the vaccine and (2) categorization of the number of farms that had bought the vaccine] show a significant association with disease levels. The categorization of the number of farms that had bought the vaccine shows a statistically significant upward trend in risk. The median farm size for each province resulted as not having a health impact on the disease risk.

(*) Test for trend: χ2 = 2.92, *P* value = 0.003.

Tables 3 shows the results of the Poisson regression models fitted to assess the association between the incidence of the disease and the exposure level to the vaccine at the provincial level.

**Table 3:** Results of the mixed-effects Poisson regression models comparing incidence rates of outbreaks

**Mixed effect model**	**Covariate**	**Exposure level**	**IRR**	**95%CI**	**AIC**
					**(fixed effects model AIC)**
**1**	Categorization of the number of farms by province that had bought the vaccine	1			133.8
**(based on modelling of crude incidence rates)**		(0-4 farms)	Referent		(140.1)
		2	1.4	0.70, 2.7	
		(5-10 farms)			
		3	4.0	1.9, 8.6	
		(>10 farms)			
**2**	Categorization of the number of farms by province that had bought the vaccine	1			
**(based on modelling of crude incidence rates)**		(0-4 farms)	Referent		132.9
		2	1.3		0.66, 2.6	(136.1)
		(5-10 farms)				
		3	3.6	1.7 , 7.5		
		(>10 farms)				
	Categorization of the number of vets or retailers by province who had bought the vaccine	1				
		(0-1 vet or retailer)	Referent			
		2	1.9	0 .91, 4.1		
		(>1 vet or retailer)				
**3**	Categorization of the number of farms by province that had bought the vaccine	1			116.3	
**(based on modelling of standardized incidence rates)**		(0-4 farms)	Referent		(114.3)	
		2	1.3	0.56, 2.9		
		(5-10 farms)				
		3	2.7	1.1, 6.5		
		(>10 farms)				
**4**	Categorization of the number of farms by province that had bought the vaccine	1			116.0	
**(based on modelling of standardized incidence rates)**		(0-4 farms)	Referent		(114.0)	
		2	1.2	0.51, 2.6		
		(5-10 farms)				
		3	2.6	1.1, 6.4		
		(>10 farms)				
	Categorization of the number of vets or retailers by province who had bought the vaccine	1				
		(0-1 vet or retailer)	Referent			
		2	1.8	0.71, 4.7		
		(>1 vet or retailer)				

AIC = Akaike Information Criterion. IRR = incidence rate ratios (IRR) indicating the increase in the risk of the disease with, respectively, the increasing number of vaccine purchaser farms or the increasing of veterinarians or retailers who had purchased the vaccine, per province. The AIC values in brackets refer to fixed-effect models based on the same covariates.

Model 1 included as independent variables the three categories of the number of farms (by province) that had purchased the vaccine. The incidence rate ratios (IRR) indicate a rise in the risk of the disease as the number of vaccine purchaser farms increases, with a 4-fold risk for level 3 (provinces where at least 10 farms had bought the vaccine). The goodness of fit of this model was better than that of the same model in which we used fixed effects, as shown by AIC values of 133.8 and 140.1, respectively, showing a significant random effect for the region.

Model 2 included as covariates both the three categories of the number of vaccine purchaser farms and the two categories of the number of veterinarians or retailers who had purchased the vaccine.

The Wald test to verify the statistical significance of the linear regression coefficient was significant in both models, evidencing a linear dose-response trend between the number of farms that had bought the vaccine and the disease incidence levels (model 1: Wald chi-square = 10.6, *P* value = 0.001; model 2: Wald chi-square = 14.3, *P* value = 0.0008).

In model 2 the risk in the provinces where more than 1 veterinarian or retailer had bought the vaccine as own stock is higher than the risk in the provinces where 0 or 1 bought it; however, the difference was not statistically significant (95% CI: 0.91 - 4.1).

Again, the comparison of the AIC of the fixed-effects model with the AIC of the mixed-effects model showed a significant random effect of the region (136.1 vs 132.9).

Model 3 and model 4 were fitted by modelling the direct standardized incidence rates. As in the case of model 1, model 3 included as an independent variable only the three categories of the number of farms (by province) that had purchased the vaccine. The incidence rate ratios (IRR) indicate that, with the increasing number of vaccine purchaser farms, the risk of the disease also increases.

As for model 2, the two categories of the number of veterinarians or retailers who had purchased the vaccine were entered into model 4 as a covariate. Again, there was an upward trend in the risk with the increasing number of farms per province that had purchased the vaccine. The risk in the provinces where more than 1 veterinarian or retailer had bought the vaccine as own stock was higher than the risk in the other provinces, however, the difference was not statistically significant.

For both models, the AIC value of the mixed-effect models was higher than the AIC value of the fixed-effects models (model 3: 116.3 vs 114.3; model 4: 116.0 vs 114.0).

The Wald test of the linear regression coefficient to check for the dose-response relationship between the three levels of vaccine exposure and scrapie incidence was 4.36 (chi-square = 0.036) for model 3 and 5.47 (chi-square 0.065) for model 4.

Finally, the population attributable fraction estimated for the number of expected cases obtained by direct standardization was 68.4%, indicating that more than two-thirds of the outbreaks observed in the study area between 2002 and 2005 might be attributable to an indirect exposure to the vaccine.

## Discussion

The findings of this study show a heterogeneous distribution of scrapie incidence in the Italian provinces between 2002 and 2005, even after standardization to account for surveillance heterogeneity. The results of the regression models suggest that some of these differences are related to the long-term effect of the circulation of a vaccine against *contagious agalactia*. This is also consistent with a linear trend, i.e., a dose-response relationship between the vaccine exposure level (number of farms that had purchased the vaccine) and the level of crude and adjusted incidence of disease. The results suggest that the effect of the vaccine is statistically significant when a large number of farms per province bought it. Finally, the very high population attributable fraction indicates that the indirect impact of vaccine circulation on the spread of scrapie within the Italian small ruminant population has been relevant in terms of secondary outbreaks likely due to the exchange of animals from directly exposed flocks.

An important aspect in epidemiological studies is the evaluation of the spatial distribution of infectious diseases because it allows potentially influencing factors to be identified. When we want to compare different geographical areas, we need to choose judiciously among the criteria for selecting outbreaks and exposed populations. In scrapie, for instance, outbreak notification relies on passive and active surveillance systems, which have been integrated in European countries since 2002. In this study we decided to not include outbreaks notified by passive surveillance because under this system the decision whether or not to include or exclude an individual animal is not under the direct control of the veterinary authorities [31]. This, in turn, can lead to underreporting of disease occurrence [18] and to differences in surveillance sensitivity in different regions.

Active surveillance coverage holds an important role in disease control and its uneven application in the field may influence the probability of case detection when a disease is present. It's possible to correct for this heterogeneity, however, by considering both the total number of animals tested for each holding and their risk category. Several studies evaluating surveillance systems and their effectiveness in public health [32-34] have highlighted their importance and complexity: to address this issue, we applied classical standardization techniques to obtain comparable incidence rates at the geographical level.

When we compared the geographical crude incidence rates, we found a regional effect on the disease distribution. This effect was no longer evident when we applied the same model to the adjusted incidence rates, suggesting that the regional effect may be connected to the applied surveillance sensitivity, which was already included in models 3 and 4 using the standardized data.

A potential source of bias in our study may be the lack of a complete database of the farms that had been exposed to the vaccine. Reconstructing the actual vaccine commercial circuit was challenging. The lists of the vaccine purchaser farms and of the seller veterinarians that were used as proxy for exposure may have been incomplete, resulting in an underestimation of the true circulation of the vaccine. On the basis of the number of vaccinated flocks in each province, we assumed that all the flocks in a given province had experienced similar exposure. Accordingly, we used average exposure levels rather than actual individual values, as is frequently done in geographical studies when the ecological fallacy is possible [35]. In both cases, however, it's unlikely that the direction of the observed impact of the vaccine on the disease distribution would have been cancelled or reversed if our data had been more complete.

The models we fitted allowed, for the first time, the quantification of the disease risk associated with the vaccine circulation also for the farms that were only indirectly exposed to it. Moreover, the estimated population attributable fraction proved a good indicator of the real impact on the entire population of vaccination. The use of this measure of association in public health can be very useful, since it allows the quantification of the cases of disease that could have been avoided after eliminating the exposure to the risk factor [29,30,36].

The very high value of population attributable fraction that we obtained may explain the scrapie incidence levels observed in the study area so far. However, it cannot completely account for the current risk of infection for the farms located in the provinces considered because other local factors will have inevitably influenced the epidemiology of the disease in the ensuing years.

The vaccine’s impact on the secondary outbreaks can be explained by the presence of additional risk factors which, in general, contribute to the spread of the disease, foremost of which is the exchange of live animals from directly exposed farms. It might be interesting to quantify the different degrees of the movement of live animals in different provinces. To do this, however, each animal would need to be individually identifiable, and such information is not currently available for sheep and goats. Furthermore, animal exchange or genetic susceptibility is unlikely to have acted as a confounding factor as there is no reason to assume that either might be linked to exposure to the vaccine.

In conclusion, with this study we were able to quantify an important long-term impact of the vaccine accident, even if the number of directly exposed farms was limited. Our study shows that a large proportion of the scrapie outbreaks, which occurred in the provinces exposed to a vaccine against *contagious agalactia* in 1997, can be explained by exposure. Therefore, relevant long-term consequences for scrapie incidence may be expected if a vaccine accident occurs. With the use of standardization techniques we can rule out a bias due to differences in surveillance sensitivity.

Beyond the effect of the vaccine, the heterogeneous distribution of scrapie in Italy deserves further research to identify local risk factors for properly assessing the effectiveness of the national control strategy of scrapie, which is largely based on the genetic selection programme.

## Endnotes

^a^ These data were aggregated at the provincial level. The territory was subdivided into provincial administrative units with their relative codes according to the 2005 data from the National Institute of Statistics [22].

## Abbreviations

TSE: Transmissible spongiform encephalopathies; IRR: Tncidence rate ratio; CI: Confidence interval.

## Competing interests

The authors declare that they have no competing interests.

## Authors’ contributions

BS, MC, IF, RG: conceived the study, participated in its design and data analysis, and drafted the manuscript. BC: was responsible for surveillance data collection and management. DR, BE, CL, CM: assisted in the data analysis and reviewed the paper. All authors have read and approved the final version of the manuscript.
